# Six-color intravital two-photon imaging of brain tumors and their dynamic microenvironment

**DOI:** 10.3389/fncel.2014.00057

**Published:** 2014-02-24

**Authors:** Clément Ricard, Franck Christian Debarbieux

**Affiliations:** ^1^Institut de Biologie du Développement de Marseille-Luminy, CNRS UMR7288 and Aix-Marseille UniversitéMarseille, France; ^2^Centre Européen de Recherche en Imagerie Médicale, Aix-Marseille UniversitéMarseille, France; ^3^Institut des Neurosciences de la Timone, CNRS UMR7289 and Aix-Marseille UniversitéMarseille, France

**Keywords:** intravital imaging, two-photon microscopy, cellular interactions, brain tumors, multicolor transgenic mouse, spectral deconvolution, cranial window

## Abstract

The majority of intravital studies on brain tumor in living animal so far rely on dual color imaging. We describe here a multiphoton imaging protocol to dynamically characterize the interactions between six cellular components in a living mouse. We applied this methodology to a clinically relevant glioblastoma multiforme (GBM) model designed in reporter mice with targeted cell populations labeled by fluorescent proteins of different colors. This model permitted us to make non-invasive longitudinal and multi-scale observations of cell-to-cell interactions. We provide examples of such 5D (x,y,z,t,color) images acquired on a daily basis from volumes of interest, covering most of the mouse parietal cortex at subcellular resolution. Spectral deconvolution allowed us to accurately separate each cell population as well as some components of the extracellular matrix. The technique represents a powerful tool for investigating how tumor progression is influenced by the interactions of tumor cells with host cells and the extracellular matrix micro-environment. It will be especially valuable for evaluating neuro-oncological drug efficacy and target specificity. The imaging protocol provided here can be easily translated to other mouse models of neuropathologies, and should also be of fundamental interest for investigations in other areas of systems biology.

## Introduction

Over the past decade, intravital two-photon microscopy (2PM) (Ustione and Piston, 2011) has become a gold standard for fundamental and pre-clinical research on various pathologies including cancer (Perry et al., 2012), auto-immune (Niesner et al., 2013) and vascular (Kuijpers and Heemskerk, 2012) diseases. It has also been applied in the field of neuroscience to study brain (Trachtenberg et al., 2002), spinal cord neuronal and vascular networks (Misgeld and Kerschensteiner, 2006; Dray et al., 2009; Fenrich et al., 2012). The technique is non-invasive and imaging can be repeated in the same animal. Chronic imaging thus avoids the sacrifice of large cohorts of animals at different time points as previously required for classical histology and immunohistochemistry experiments (Zhai et al., 2011; Piao et al., 2012). Thanks to its high spatial resolution, dynamic subcellular observations can be performed in the brain of living transgenic mice (e.g., Thy1-CFP, Feng et al., 2000). Exogenous fluorescent markers can also be used to label specific compartments such as blood vessels through intravenous injections of Rhodamine-dextran (Verant et al., 2008; Ricard et al., 2009). The outstanding imaging depth of 2PM in scattering media permits investigators to scan most of the subdural brain cortical layers in mice (Helmchen and Denk, 2005; Kobat et al., 2009). In the case of brain tumors, it offers unique means for studying the kinetics of tumor cell infiltration/migration as well as permitting access to correlated successive events such as vascular remodeling induced by tumor cell proliferation. The efficacy of anti-tumor and anti-angiogenic drugs can thus be assessed and their action mechanism clarified (Von Baumgarten et al., 2011; Ricard et al., 2013).

The role of the tumor microenvironment has recently become a hot topic in neuro-oncology (Charles et al., 2011; Hanahan and Weinberg, 2011). The microenvironment includes several populations of cells that interact directly with extracellular matrix proteins. All these components should ideally be monitored simultaneously to elucidate the key mechanisms underlying tumor progression. However, current imaging protocols are designed to study at most 3 parameters in the same intravital imaging sessions, and are usually limited to the study of tumor cells and vasculature only (Winkler et al., 2009; Von Baumgarten et al., 2011). Glioblastoma multiforme (GBM) is the most aggressive form of brain tumor, with poor prognosis and no curative treatment available to date (Deangelis, 2001; Prados et al., 2004; Ricard et al., 2012b). We have therefore developed an orthotopic GBM murine model, consisting of the graft of fluorescently-labeled tumor cell spheroids into the cerebral cortex of multicolor fluorescent transgenic mice where each cell population and compartment of interest fluoresce in a specific color. We have subsequently set up dedicated 2PM and image processing protocols to routinely observe 6 components on a daily basis in the same living animal without side effects. We describe the complete methodology and illustrate new dynamic information that can be obtained at micro and macroscopic scales with imaging sessions lasting 1 h on average. Finally, we discuss the possible implications of this approach for pre-clinical studies.

## Biological methods

### Animal care guidelines

All experimental procedures were performed in accordance with the French legislation and in compliance with the European Community Council Directive of November 24, 1986 (86/609/EEC) for the care and use of laboratory animals. The research on animals was authorized by the Direction Départementale des Services Vétérinaires des Bouches-du-Rhône (license D-13-055-21) and approved by the National Committee for Ethic in Animal Experimentation (Section N°14; project 87-04122012).

### Cell culture

Cells from the murine GL261 GBM cell line (Newcomb and Zagzag, 2009), were transfected with a plasmid encoding for DsRed (pDsRed2-N1, Clontech, Mountain View, USA). Cells that stably express DsRed were selected using Geneticin (0.5 mg/ml, Gibco) and cultured as monolayers in RPMI1640 + GlutaMAX-1 (Gibco 61870) supplemented with 10% Fetal Calf Serum (Thermo Scientific) and Geneticin (0.5 mg/ml,Gibco). Cells were kept at 37°C in a 5% CO_2_ atmosphere. Confluent cells were plated on Petri dishes coated with 1% soft agarose to induce spheroid formation.

### Transgenic mice

Connexin43-CFP (astrocytes Degen et al., 2012), Thy1-GFP (neurons Feng et al., 2000) and CD11c-YFP (reactive microglia and dendritic cells Lindquist et al., 2004) mice were crossed to obtain the triple transgenic mouse strain, Connexin43-CFP/Thy1-GFP/CD11c-YFP, used in our experiments.

### Glioblastoma animal model

The technology had been described in detail in Ricard et al. (2013). Briefly, adult mice (>7 weeks) were anaesthetized by an intraperitoneal injection of a mixture of Xylazine/Ketamine (10 mg/kg and 100 mg/kg, respectively) and positioned on a stereotactic frame. A 3–4 mm diameter craniotomy was performed on the left parietal bone. The dura-mater was incised by a 31G needle and a 250 μm spheroid of GL261-DsRed murine GBM cells was then injected into the cerebral cortex approximately 250 μm below the brain surface. A Sephadex hemi-bead with a diameter that fit through the dura-mater opening was inserted in the injection wound and glued using histo-compatible acrylic glue (Cyanolit). A round glass coverslip (5 mm diameter) was then sealed on the adjacent bone and fixed to the skull by dental cement. Animals were allowed to recover for 15 days post-surgery before the first imaging session.

### Animal preparation for imaging

Prior to each imaging session, mice were anaesthetized by an intraperitoneal injection of a mixture of Xylazine/Ketamine (10 mg/kg and 1.0 mg/kg, respectively), injected intravenously with 100 μl of a Cascade Blue conjugate dextran (70 kDa) solution (20 mg/ml in phosphate saline buffer (Sigma)) and positioned on a stereotactic frame. Each imaging session, including anesthesia induction, animal positioning and acquisition lasted approximately 45 min–1 h.

## Experimental setup and image processing

**Figure 1:**
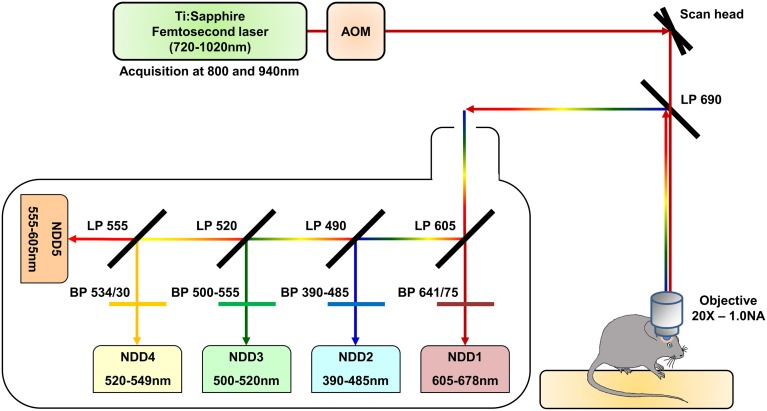
**Schematic representation of the two-photon microscopy setup**. The excitation beam is produced by a femtosecond pulsed infrared tunable (720–1020 nm) laser (Mai-Tai, Spectra Physics). The laser power is modulated by an Acousto-Optic Modulator (AOM). The beam is scanned in the xy direction by galvanometric mirrors present in the scan head of a Zeiss LMS 7 MP two-photon microscope. The beam then passes through a LP690 dichroic mirror and is focused in the brain of the anaesthetized animal by a 20X-1.0 NA water immersion objective. The emitted epifluorescence is collected and reflected by the LP690 mirror in a non-descanned mode. The fluorescence is finally splitted and filtered using a set of dichroic mirrors and filters and collected by a set of 5 non-descanned detectors mounted in cascade (NDD). The characteristics of the dichroic mirrors and filters are depicted on the scheme.

We used a Zeiss LSM 7 MP two-photon microscope home-modified to allow animal positioning below the 20X water immersion objective (1.0 NA) and coupled to a femtosecond pulsed infrared tunable laser (Mai-Tai, Spectra Physics). After two-photon excitation, epifluorescence signals were collected and separated by dichroic mirrors and filters on 5 independent non-descanned detectors (NDD) (Figure 1). Images were acquired sequentially using an excitation wavelength tuned at 800 nm and then at 940 nm, and were assembled into a spectral hyperstack. Gains and offsets were identical for all the detectors, except for the red channels whose gain was slightly reduced to compensate for the very strong expression of DsRed in tumor cells.

**Figure 2:**
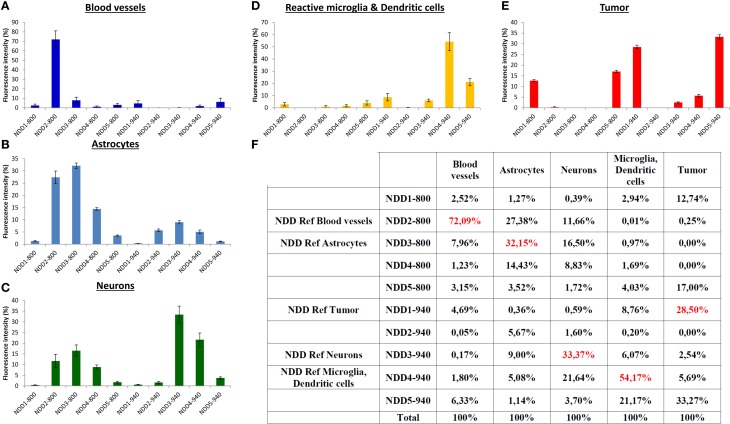
**Normalized spectral signatures of each biological element on the non-descanned detectors array**. **(A–E)** normalized (%) contribution of each element on the NDDs for excitations at 800 nm (NDD1-800–NDD5-800) and 940 nm (NDD1-940–NDD5-940). **(F)** Numerical values (%) used for spectral deconvolution (red: contribution of the element of interest in its reference NDD).

Each fluorescent reporter (*i* = 1–5) used to selectively label a physiological component exhibited a footprint on all detectors (NDD1-800–NDD5-800, numbered #1 to #5 and NDD1-940 – NDD5-940, #6 to #10). Its spectral signature on the detection arrays was represented by a normalized vector *S*_i_ = [*C*_i,1_, …, *C*_i,10_] whose coordinates *C_i,k_* (*k* = 1–10) corresponded to the relative contribution of each detector k to the sum of intensities measured on all detectors (in percent, Figure 2). Spectral signatures were determined from regions of interest (ROIs, *n* = 5) selected manually for their unambiguous and exclusive expression of one of the fluorescent labels of interest. Control intensities measured in a ROI not containing any fluorescent object were systematically subtracted from the signals of fluorescent ROIs in order to correct for background signals prior to normalization. For each fluorophore (i), the main contribution to the global fluorescence was identified as *C*_i,REFi._ and the detector #REFi was thus defined as the reference detector to outline biological objects labeled with the fluorophore (i). Quantitative data are depicted in Figure 2F.

Spectral deconvolution of the channel #REFi was finally obtained by subtracting the minor contributions of all other fluorophores in the channel #REFi. Corrections for each fluorophores j were then expressed as percentages of the fluorescence intensities measured on their respective reference detector #REFj.

After determining the spectral signature *S_i_* of the fluorophore of interest and the signatures S_j_ for all other contaminating labels in ROIs selectively labeled with one fluorophore,

$$\begin{array}{ll} S_{\text{i}}=\left(C_{\text{i},1},...,C_{\text{i},{\text{ REF}}_{\text{i}}},\;C_{\text{i,10}}\right) & \text{with }1\leq {\text{REF}}_{\text{i}}\leq 10\text{ and}\\ S_{\text{j}}=\left(C_{\text{j},1},...,C_{\text{j},{\text{ REF}}_{\text{j}}},\;C_{\text{j,10}}\right) & 1\leq {\text{REF}}_{\text{j}}\leq 10 \end{array}$$

spectral deconvolution of the whole image was obtained using the following equation for each of its pixels:

$$I_{\text{i},\text{Deconv}}=I_{\text{i},\text{REF}}-\sum_{\text{j}\neq \text{i}}\alpha_{\text{j}}\cdot I_{\text{j},\text{REF}}$$

where: *I*_i,Deconv_ is the spectrally unmixed image for element *i*. where: *I*_i,REF_ is the reference image for element *i*; it displays the strongest fluorescent signal contribution for the element *i*. where: *I*_j,REF_ is the reference image for element *j*; it displays the strongest fluorescent signal contribution for the element *j*. and:

$$\alpha_{\text{j}}=\frac{C_{\text{j},\text{REFi}}}{C_{\text{j},\text{REFj}}}$$

where: *C*_*j*,REFi_ is the relative contribution of element *j* on the reference NDD for element *i*. It can be read from *S*_j_; where: *C*_*j*,REFj_ is the relative contribution of element *j* in its own reference NDD. It can be read from *S*_j_.

Spectral deconvolution was performed for the 5 fluorophores (CFP, GFP, YFP, DsRed, cascade blue) to generate a set of five image stacks representative of the distribution of the following components: astrocytes, neurons, CD11c-positive cells, tumor and vasculature. A sixth element, SHG arising from dura-mater and peritumoral collagen, was only present in the superficial layers of the brain and was spectrally unmixed from NDD2-940 using the same approach (magenta pseudo color in Figure 4). All spectrally unmixed individual images for each biological component and the collagen images were finally merged together to obtain a corrected 6-color image (blue: vasculature, cyan: astrocytes, green: neurons, yellow: CD11c-positive immune cells, red: tumor, magenta: collagen). Custom-written ImageJ macros were used to spectrally unmix the fluorescent signals.

## Results and discussions

A set of five detectors was installed in the non-descanned position of our 2-photon upright microscope. Filter cubes were set in order to analyze the intensity of the emitted fluorescence in five non-overlapping bands covering most of the visible light spectrum. Bands were selected to include the acknowledged emission peak for the most classical fluorophores (Figure 1). The fluorescent signal arising from the different fluorophores was then collected on these 5 NDDs under two different excitation conditions, sequentially using excitation wavelengths at 800 nm and 940 nm. The contribution of each fluorescently labeled component on the 10 NDDs (5 from the 800 nm excitation and 5 from the 940 nm excitation) is depicted in Figure 2. It should be noted that the background signal has to be measured on each NDD and subtracted from the fluorophore signal acquired. The background signal arises both from electronic and shot noise but also from brain autofluorescence. Contribution of the autofluorescence to the fluorescence signal depends both on the expression level of the fluorescence probe and on its relative brightness. It is expected to be larger for thin objects whose size is below the voxel resolution. Comparison between raw and background subtracted images indicated that endofluorescence accounted for 10–15% of the reference fluorescence signal for the dendritic processes and 7–10% for somas in our mice (Supplementary Figure 1). The spectral signature of the background and of the fluorophores is moreover expected to vary according to the imaging depth due to differential scattering and absorption. Variations due to depth were limited to less than 15% over the first 300 μm below the dura (Supplementary Figure 2). Imaging at greater depths would require measurement of the spectral signature of used fluorophores at various depths to optimize spectral unmixing. For each biological component, the NDD recording the highest relative signal was defined as the Reference NDD for this component: NDD2-800 for vasculature (Cascade-Blue), NDD3-800 for astrocytes, NDD1-940 for tumor, NDD3-940 for neurons and NDD4-940 for CD11c-positive cells. For tumor cells, although the contribution was slightly higher in NDD5-940 than in NDD1-940 (33.27 vs. 28.50%, Figure 2F), the latter was preferred as it minimized the contaminations by other fluorophores. A sixth signal corresponding to the second harmonic generation reflected by the dura-mater and peritumoral collagen was analyzed on detector NDD2-940.

**Figure 3:**
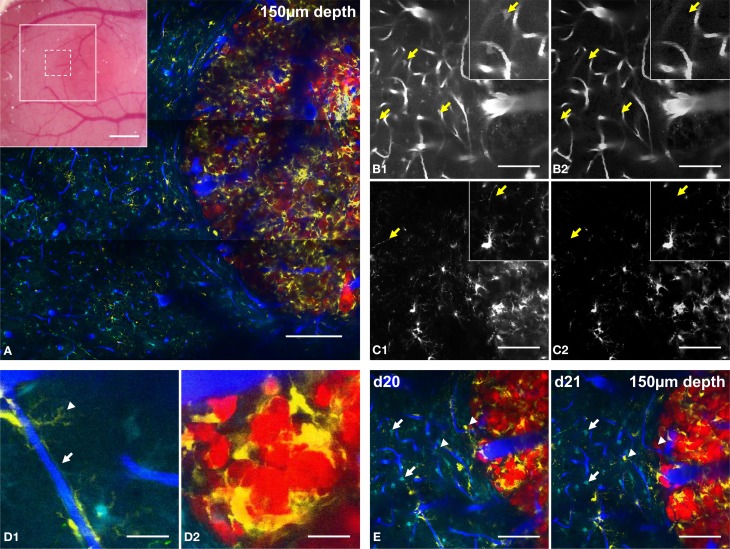
**Five-color intravital two-photon imaging of glioblastoma tumor and its dynamic environment**. **(A)** 5-colors tile scan (3 × 3 images) of the GBM and its microenvironment; inset: macroscopic image of the cranial window with the area covered by the tile scan (large white square) and the area covered by a single plane acquisition (small dotted square). **(B)** Image of vasculature taken from NDD2-800 before **(B1)** and after **(B2)** spectral deconvolution; arrows: location of identified astrocytes that are removed from the image after spectal deconvolution; inset: zoom (100 × 100 μm). **(C)** Image of CD11c-positive cells taken from NDD4-940 before **(C1)** and after **(C2)** spectral deconvolution; arrows: location of identified neurons that are removed after spectal deconvolution; inset: zoom (100 × 100 μm). Note that the background generated by DsRed positive tumor cells is removed from the image **(D)**. Examples of cellular interactions at the level of their fine processes. **(D1)** zoom of a CD11c-positive cell wrapping its processes around a blood vessel (arrowhead) and of an astrocyte whose processes contact the vessel walls as part of the blood-brain-barrier (arrow); **(D2)**: zoom of CD11c-positive cells surrounding tumor cells in the tumor core. **(E)** Same field of view at day 20 (d20) and day 21 (d21) post-implantation; note that astrocytes are stable (arrows) while CD11c-positive cells appear or disappear (arrowheads). Colors: blue: vasculature, cyan: astrocytes, green: neurons, yellow: CD11c-positive cells, red: tumor cells. Scale bars: **(A)**: 200 μm (inset: 500 μm), **(B–E)**: 100 μm. All images were taken at 150 μm depth.

Spectral unmixing was further improved by subtracting the signals of contaminating fluorophores on the Reference NDD of each biological component (see Methods). For example, the significant contribution of astrocyte signals in the reference NDD for vasculature (NDD2-800) was removed (arrows in Figure 3B) as well as the contribution of neurons (arrows in Figure 3C) in the reference NDD for CD11c-positive cells (NDD4-940). Alternatively, deconvolution algorithms available as free ImageJ plugins can also be applied (Joachim Walter's Spectral Unmixing plugin based on Zimmermann et al., 2002; Neher and Neher, 2004).

The combination of multichannel/multiexcitation 2PM and spectral unmixing methods therefore facilitates detailed micrometric characterizations of the cellular processes of various cell types. In the context of our GBM model, the fine processes of CD11c-positive immune cells and connexin–positive astrocytes could be independently visualized along the blood vessel walls. This supports the viewer that the latter actively contribute to the blood-brain-barrier formation (Abbott et al., 2006) (Figure 3D1, arrowhead and arrow, respectively). Close apposition and interaction between CD11c-positive cells and tumor cell bodies were also highlighted inside the tumor core (Figure 3D2) consistent with the involvement of CD11c-positive antigen presenting cells as part of the immune response that fights against tumor progression (Prins et al., 2011; Gabrilovich et al., 2012).

**Figure 4:**
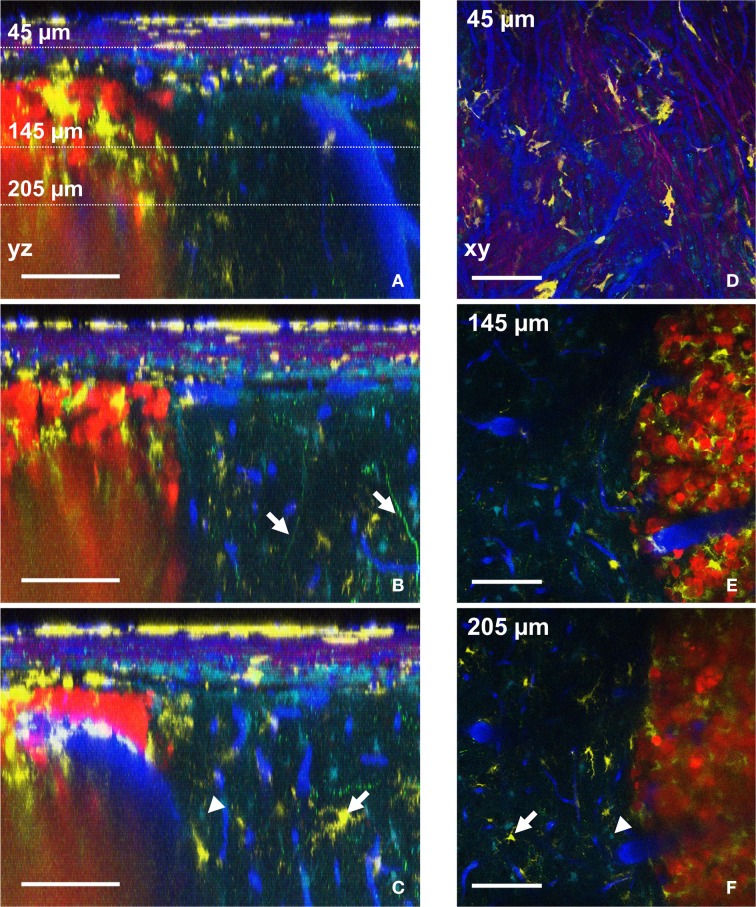
**Distributions of six components assessed by intravital two-photon imaging**. **(A–C)** Orthogonal reconstructions obtained from a stack acquired from 0 to 300 μm below the glass coverslip with a z-step of 3 μm. Each YZ image shows the maximum intensity projection over 10 microns along the X axis. **(A)** Note the vertical orientation of major brain vessels; dotted lines: the levels of xy-sections are shown in **D–F**. **(B)** Apical dendrites of neurons are visible (arrows). **(C)** Astrocyte (arrowhead) and CD11c-positive cell (arrow) also visible in **F**. **(D–F)** xy-sections taken at 45 μm **(D)**, 145 μm **(E)**, and 205 μm **(F)** below the glass coverslip. **(D)** Note the SHG signal (magenta) corresponding to the collagen fibers at the level of the dura-mater. **(E)** CD11c-positive cells have invaded the tumor but not surrounding healthy tissues. **(F)** Astrocyte (arrowhead) and CD11c-positive cell (arrow) also visible in **(C)**. Colors: blue: vasculature, cyan: astrocytes, green: neurons, yellow: CD11c-positive cells, magenta: SHG signal (dura-mater), red: tumor cells. Scale bars: 100 μm.

The possibility to compute orthogonal reconstructions from XY images acquired at different depths in the brain of the anesthetized animal provides access to morphological information that would be lost with classical postmortem histology techniques. In our experimental conditions, the vertical branching pattern of blood vessels (Figure 4A, right of the picture) and the track used by apical dendrites of layer 4–5 pyramidal neurons (Figure 4B, arrows) could both be followed reliably. Something that is normally difficult to visualize in fixed thin serial sections. It should be noted that acquisition parameters were set such that the fine processes of Thy1-GFP cells (neurons) could be readily visualized at the expense of cell bodies, which were saturated and could therefore not be properly unmixed. Nevertheless, cell bodies of neurons could be qualitatively differentiated from astrocytes somata (Supplementary Figure 3). If required, different acquisition parameters can be chosen that optimize imaging of cell bodies rather than fine processes. The progressive infiltration of CD11c-positive cells along the dorso-ventral axis can similarly be highlighted in orthogonal images (Figures 4C,F, arrows). Accumulation of CD11c-positive cells was pre-dominantly found inside the tumor while relatively low numbers of these cells were found in the surrounding brain parenchyma (Figures 4E,F). The in-depth lateral spatial resolution deteriorates faster inside the tumor than in surrounding healthy tissues due to the highly scattering properties of the dense and structurally disorganized tumor tissue (Figures 4A–C,E,F). A more powerful femtosecond pulsed laser in less diffusible spectral range (Kobat et al., 2009) (few watts and above 940 nm) aiming at efficient two-photon excitation of red-shifted fluorophores and triphoton excitation of blue shifted ones, might be used to overcome this limitation.

Spectral deconvolution of the signals from NDD2-940 outlines the superficial collagen fibers (Figures 4A–C) and reveals their privileged orientation (Figure 4D). SHG signals from collagen fibers(Cicchi et al., 2013) of the dura-mater could only be visualized in the most superficial 50 μm below the glass coverslip (Figure 4D), due to the facts that reflected SHG signals are intrinsically smaller than transmitted ones. Any generated blue photons are easily scattered and absorbed by biological tissue.

The 20X objective used for this study gave access to a field of view of 400 × 400 microns. Larger fields of view covering several millimeters were acquired by tiling several elementary fields (Figure 3A). Such microscopic imaging of macroscopic fields of view provides unique pre-clinical information about the structural heterogeneities of tumor vessels and intratumoral immune cell distributions relatively to healthy brain regions. Because two sets of images have to be acquired at two different excitation wavelengths at a 2 hz acquisition rate for 512 × 512 pixels images, approximately 40 min were required to acquire large field of view recordings as presented in Figure 3A (9 fields of view with 3 micron z steps over 400 microns depth). The non-invasiveness of 2PM allows for repeated imaging of the same areas in order to study tumor progression and micro-environmental dynamics on a daily basis. Repeated imaging of tumor burden on day 20 and day 21 post-surgery (Figure 3E), for example, revealed the dynamic distribution of immune CD11c-positive cells (arrowheads) while astrocytes, in contrast did not move (arrows).

**Figure 5:**
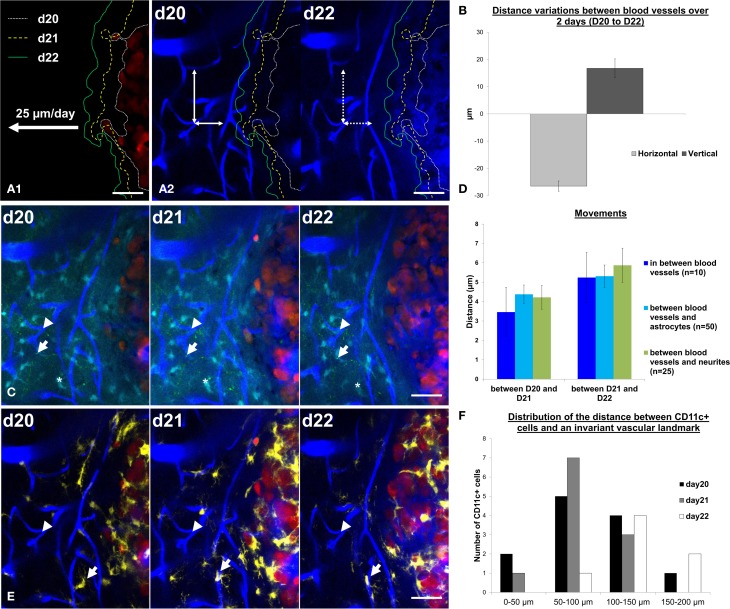
**Cell dynamics and deformations due to tumor growth can be tracked over time**. **(A1)** Tumor margin are highlighted at day 20 (d20, white small dotted line), day 21 (d21, yellow dotted line) and day 22 (d22, green line). Tumor expansion rates were measured at 25 ± 2 μm/day. **(A2)** Tumor margins at d20, d21, and d22 were overlaid on vasculature images at day 20 and day 22. Notice the decrease of the distances between blood vessels in the horizontal direction (in the direction of tumor growth, horizontal double-arrow) and an increase in the vertical direction (perpendicular to the direction of tumor growth, vertical double-arrow). Movements between d20 and d22 are highlighted by the mismatch between arrowheads and vessel walls (dotted line on d22). **(B)** Quantification of the distance variation (μm) between blood vessels in the horizontal direction (in the direction of tumor growth, light gray) and in the vertical direction (perpendicular to the direction of tumor growth, dark gray). **(C)** z-projection of 6 slices over 15 μm in the same field of view at day 20, day 21, and day 22 on a xyz registered image. Vascular landmarks (blue, arrowhead), astrocytes (cyan, asterisk) and neurites (green dots, arrow) appear stable over the observation period. **(D)** Quantification of the movements (between d20 and d21, left panel and between d21 and d22, right panel) between blood vessels (blue, *n* = 10 measurements), of astrocytes relative to blood vessels (cyan, *n* = 50 measurements) and of neurites relative to blood vessels (green, *n* = 25 measurements). **(E)** Same field of view as in C (z-projection of 6 slices over 15 μm) at day 20, day 21, and day 22 on a xyz registered image showing only the CD11c (yellow), blood vessels (blue) and tumor (red) channels for the sake of clarity. Note the dynamics of one CD11c-positive cell identified by an arrow. **(F)** Frequency distribution of the distances to peritumoral CD11c-positive cell somas relative to a reference vascular landmark (arrowhead in **E**) at day 20, day 21, and day 22. Colors: blue: vasculature, cyan: astrocytes, green: neurons, yellow: CD11c-positive cells, red: tumor cells. Scale bars: 50 μm.

Such information is of interest given the strong influence that the micro-environment has on GBM development (Charles et al., 2011; Hanahan and Weinberg, 2011). Multiparametric dynamic imaging of the tumor and its micro-environment provide unique data that could benefit our understanding of current models of tumor progression and identify new therapeutic targets. Whereas dynamic monocolor imaging allowed for quantification of the daily tumor progression rate (Figure 5A1), dual color imaging of blood vessels and tumor cells further highlighted the influence of tumor growth on the peritumoral vessels (Figure 5A2). In the tumor margin, the distances between vascular branches increased over time along the vertical axis while they shrunk along the horizontal axis (Figure 5B). These changes could result either from vascular remodeling as observed in peritumoral areas for other tumor models (Ricard et al., 2013), or from the compression exerted by the expanding tumor onto the tissue. To discriminate between the two hypotheses, we here used information from the color channels corresponding to astrocytes and neurons. The displacements of identified astrocytes or neurons relative to identified vessel branches exhibited amplitudes similar to the movements of the vessels themselves (Figure 5D) as expected from a global deformation of all the cellular components. Over 3 days, deformation was restricted to a few microns in amplitude, indicating that the tumor barely influenced the structure of the parenchyma beyond 50 microns away from its invasive margin (Figure 5C). Inflammatory cells represented the most dynamic features of the pathological brain as illustrated by their quickly changing densities and the distribution patterns of yellow Cd11c(+) cells (Figure 5F) inside stable neuroglial and vascular meshworks (Figures 5C,E).

Since this approach can be adapted to study other cell populations or cell interactions with extracellular matrix components using relevant strains of transgenic fluorescent animals, we believe that our setup, protocol and animal model open new perspectives in the field of neuro-oncology. Generally speaking, the multiexcitation multicolor approach described here can be applied to study many pathologies including spinal cord lesion (Fenrich et al., 2012) but also diseases of other organs such as intestines (Ricard et al., 2012a; Zhuo et al., 2012), liver (Honda et al., 2013) or lungs (Fiole et al., 2012). Importantly, this multiplexed approach should prove valuable for pharmaceutical research since the effect of candidate therapeutical molecules can be simultaneously assessed *in vivo*, on several cellular targets. In addition to a reduction in the use of experimental animals, the current methods provide a powerful tool for studying drug specificity and the optimal time windows for treatment.

## Author contributions

Clément Ricard and Franck Christian Debarbieux designed, performed the experiments, the analysis; Clément Ricard and Franck Christian Debarbieux wrote the manuscript; Franck Christian Debarbieux obtained the funding.

### Conflict of interest statement

The authors declare that the research was conducted in the absence of any commercial or financial relationships that could be construed as a potential conflict of interest.

## Supplementary material

Supplementary Figure 1 **Astrocytes soma and process spectral signatures**. Normalized (%) contribution of astrocytes soma (top panel) and process (middle panel) on the NDDs for excitations at 800 nm (NDD1-800–NDD5-800) and 940 nm (NDD1-940–NDD5-940) without (left) and with (right) background subtraction. All measurements were performed at 100 μm below the dura-mater. Measurements were realized on 5 different ROI and error bars are s.e.m.

Supplementary Figure 2 **Astrocytes spectral signature at 50, 150, and 250 μm below the dura-mater**. **(A)** Normalized (%) contribution of astrocytes on the NDDs for excitations at 800nm (NDD1-800–NDD5-800) and 940 nm (NDD1-940–NDD5-940) at 50, 150, and 250 μm below the dura-mater without (top panel) and with (bottom panel) background subtraction. **(B)** Measurements were realized on 5 different ROI and error bars are s.e.m.

Supplementary Figure 3 **Neuron and astrocytes somas after spectral unmixing**. Maximum intensity projection of 5 images with a z-space of 3 μm acquired at 200 μm below the dura-mater. Spectral unmixing was performed with the parameters presented in Figure 2. Acquisition parameters were set to highlight the fine neuritic processes which hence led to a saturation of the neuron soma. Nevertheless cell bodies from neuron (arrow) and astrocytes (arrowheads) can be easily discriminated after spectral unmixing. Colors: blue: vasculature, cyan: astrocytes, green: neurons. Scale bar: 100 μm.
