# Supplementary material for: Six-color intravital two-photon imaging of brain tumors and their dynamic microenvironment
Source: Front Cell Neurosci. 2014 Feb 24;8:57. doi: 10.3389/fncel.2014.00057 (PMC3932518; doi:10.3389/fncel.2014.00057)

Astrocytes – 100µm depth

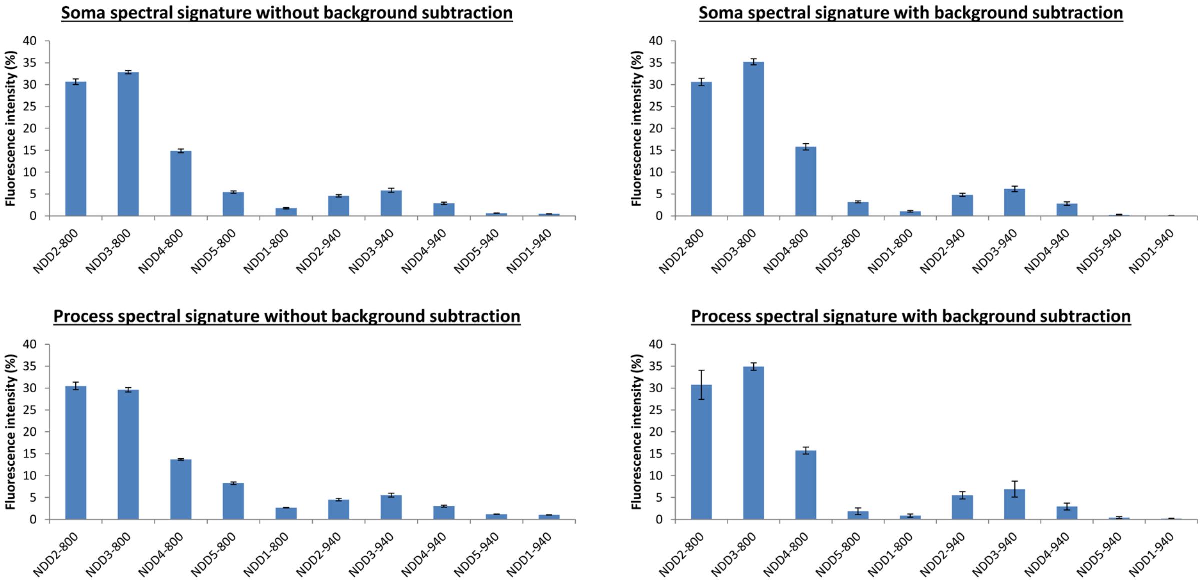

Supplement: Supplementary Figure 1 — Astrocytes soma and process spectral signatures. Normalized (%) contribution of astrocytes soma (top panel) and process (middle panel) on the NDDs for excitations at 800 nm (NDD1-800–NDD5-800) and 940 nm (NDD1-940–NDD5-940) without (left) and with (right) background subtraction. All measurements were performed at 100 μm below the dura-mater. Measurements were realized on 5 different ROI and error bars are s.e.m. [file Presentation1.PDF]
