# Supplementary material for: Six-color intravital two-photon imaging of brain tumors and their dynamic microenvironment
Source: Front Cell Neurosci. 2014 Feb 24;8:57. doi: 10.3389/fncel.2014.00057 (PMC3932518; doi:10.3389/fncel.2014.00057)
Supplement: Supplementary Figure 2 — Astrocytes spectral signature at 50, 150, and 250 μm below the dura-mater. (A) Normalized (%) contribution of astrocytes on the NDDs for excitations at 800nm (NDD1-800–NDD5-800) and 940 nm (NDD1-940–NDD5-940) at 50, 150, and 250 μm below the dura-mater without (top panel) and with (bottom panel) background subtraction. (B) Measurements were realized on 5 different ROI and error bars are s.e.m. [file Presentation2.PDF]

# Astrocytes

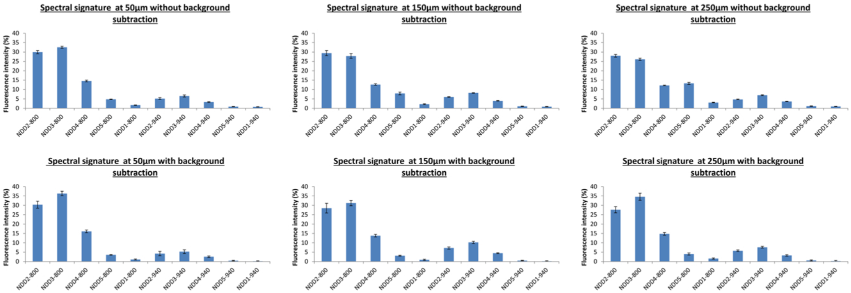
Spectral signature at 50µm without background subtraction
Spectral signature at 150µm without background subtraction
Spectral signature at 250µm without background subtraction
Spectral signature at 50µm with background subtraction
Spectral signature at 150µm with background subtraction
Spectral signature at 250µm with background subtraction
Fluorescence intensity (%)
0
5
10
15
20
25
30
35
40
NDD2-800
NDD3-800
NDD4-800
NDD5-800
NDD1-800
NDD2-940
NDD3-940
NDD4-940
NDD5-940
NDD1-940
