# Supplementary figures and images for: Six-color intravital two-photon imaging of brain tumors and their dynamic microenvironment
Source: Front Cell Neurosci. 2014 Feb 24;8:57. doi: 10.3389/fncel.2014.00057 (PMC3932518; doi:10.3389/fncel.2014.00057)

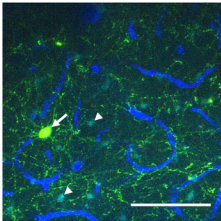

Supplement: Supplementary Figure 3 — Neuron and astrocytes somas after spectral unmixing. Maximum intensity projection of 5 images with a z-space of 3 μm acquired at 200 μm below the dura-mater. Spectral unmixing was performed with the parameters presented in Figure 2. Acquisition parameters were set to highlight the fine neuritic processes which hence led to a saturation of the neuron soma. Nevertheless cell bodies from neuron (arrow) and astrocytes (arrowheads) can be easily discriminated after spectral unmixing. Colors: blue: vasculature, cyan: astrocytes, green: neurons. Scale bar: 100 μm. [file Presentation3.PDF]
